# Phase III Pivotal comparative clinical trial of intranasal (iNCOVACC) and intramuscular COVID 19 vaccine (Covaxin^®^)

**DOI:** 10.1038/s41541-023-00717-8

**Published:** 2023-08-18

**Authors:** Chandramani Singh, Savita Verma, Prabhakar Reddy, Michael S. Diamond, David T. Curiel, Chintan Patel, Manish Kumar Jain, Sagar Vivek Redkar, Amit Suresh Bhate, Vivek Gundappa, Rambabu Konatham, Leelabati Toppo, Aniket Chandrakant Joshi, Jitendra Singh Kushwaha, Ajit Pratap Singh, Shilpa Bawankule, Raches Ella, Sai Prasad, Brunda Ganneru, Siddharth Reddy Chiteti, Sreenivas Kataram, Krishna Mohan Vadrevu

**Affiliations:** 1 All India Institute of Medical Sciences, Patna, Bihar India; 2 Pt. BD Sharma Postgraduate Institute of Medical Sciences (PGIMS), Rohtak, Haryana India; 3 Nizams Institute of Medical Sciences, Hyderabad, Telangana India; 4 Department of Medicine, Molecular Microbiology, Pathology & Immunology, Washington University School of Medicine, St. Louis, MO USA; 5 Department of Radiation Oncology, Washington University School of Medicine, St. Louis, MO USA; 6 Aatman Hospital, Ahmedabad, India; 7 Maharaja Agrasen Super Speciality Hospital, Jaipur, Rajasthan India; 8 Redkar Hospital and Research Centre, Goa, India; 9 Jeevan Rekha Hospital, Belgaum, Karnataka India; 10 Rajarajeswari Medical College and Hospital, Bangalore, Karnataka India; 11 Visakha Institute of Medical Science, Visakhapatnam, Andhra Pradesh India; 12 Malla Reddy Narayana Multispeciality Hospital, Hyderabad, Telangana India; 13 Oyster and Pearl Hospitals (Phadnis Clinic), Pune, Maharashtra India; 14 Prakhar Hospital, Kanpur, Uttar Pradesh India; 15 Rana Hospital, Gorakhpur, Uttar Pradesh India; 16 Acharya Vinobha Bhave Rural Hospital, Wardha, Maharashtra India; 17 Bharat Biotech International Limited, Hyderabad, India

**Keywords:** Translational research, Viral infection

## Abstract

One of the most preferable characteristics for a COVID-19 vaccine candidate is the ability to reduce transmission and infection of SARS-CoV-2, in addition to disease prevention. Unlike intramuscular vaccines, intranasal COVID-19 vaccines may offer this by generating mucosal immunity. In this open-label, randomised, multicentre, phase 3 clinical trial (CTRI/2022/02/40065; ClinicalTrials.gov: NCT05522335), healthy adults were randomised to receive two doses, 28 days apart, of either intranasal adenoviral vectored SARS-CoV-2 vaccine (BBV154) or licensed intramuscular vaccine, Covaxin^®^. Between April 16 and June 4, 2022, we enrolled 3160 subjects of whom, 2971 received 2 doses of BBV154 and 161 received Covaxin. On Day 42, 14 days after the second dose, BBV154 induced significant serum neutralization antibody titers against the ancestral (Wuhan) virus, which met the pre-defined superiority criterion for BBV154 over Covaxin^®^. Further, both vaccines showed cross protection against Omicron BA.5 variant. Salivary IgA titers were found to be higher in BBV154. In addition, extensive evaluation of T cell immunity revealed comparable responses in both cohorts due to prior infection. However, BBV154 showed significantly more ancestral specific IgA-secreting plasmablasts, post vaccination, whereas Covaxin recipients showed significant Omicron specific IgA-secreting plasmablasts only at day 42. Both vaccines were well tolerated. Overall reported solicited reactions were 6.9% and 25.5% and unsolicited reactions were 1.2% and 3.1% in BBV154 and Covaxin^®^ participants respectively.

## Introduction

Although the incidence of COVID-19 cases and deaths are substantially reduced globally than at the pandemic’s peak, the continuing emergence of SARS-CoV-2 variants of concern (VOCs), including the most recent highly transmissible Omicron variants mean the threat of the pandemic is not over^1,2^. Therefore, efforts to provide protection through prophylactic vaccination must continue. However, all currently licensed injectable SARS-CoV-2 vaccines have diminished efficacy against emerging VOCs, whose mutated forms of S-protein make them less vaccine-sensitive^3,4^. In addition, there is a limited vaccine efficacy against asymptomatic infection and transmission of emerging variants^5^. The nasal mucosa is the first anatomical and immunological barrier the SARS-CoV-2 virus must overcome to induce infection^6^. Current intramuscular COVID-19 vaccines are designed to elicit robust systemic immunity but induce limited mucosal immunity, which may be critical to block SARS-CoV-2 infection and transmission that allow breakthrough infections in fully vaccinated individuals^7,8^. By producing both mucosal protective immunity at the site of infection and systemic immunity, an intranasal vaccine may offer the advantage of being efficacious against the disease and infection, while also decreasing transmission^9^.

Bharat Biotech International Limited (BBIL), India, has developed BBV154, a chimpanzee adenoviral-vectored SARS-CoV-2 intranasal vaccine encoding a prefusion-stabilized spike protein with two proline substitutions in the S2 subunit (GenBank: QJQ84760.1)^10^. In preclinical studies conducted in mice, rats, hamsters, and rabbits, BBV154 elicited robust mucosal and systemic humoral and cell-mediated responses^11^. In a SARS-CoV-2 challenge model using the highly susceptible K18-hACE2 transgenic mouse, one intranasal dose of BBV154 conferred superior immunity compared with one or two intramuscular immunizations with the same dose of the same vaccine^10^. Furthermore, studies conducted in K18-hACE2 transgenic mice, Syrian golden hamsters, and rhesus macaques, an intranasal dose of BBV154 prevented upper and lower respiratory tract infections and inflammation due to SARS-CoV-2^12,13^.

Following the demonstration of the safety and immunogenicity of BBV154 in phase 1 and 2 clinical trials in humans (unpublished data, Supplementary Table 4), we now report the interim immunogenicity and safety findings from a phase 3, controlled, randomised, open-label trial of BBV154, when administered as a homologous primary vaccination series comprising two doses given four weeks apart. This study includes a lot-to-lot consistency assessment of three consecutive manufacturing lots for regulatory authorities.

## Results

**Fig. 1:**
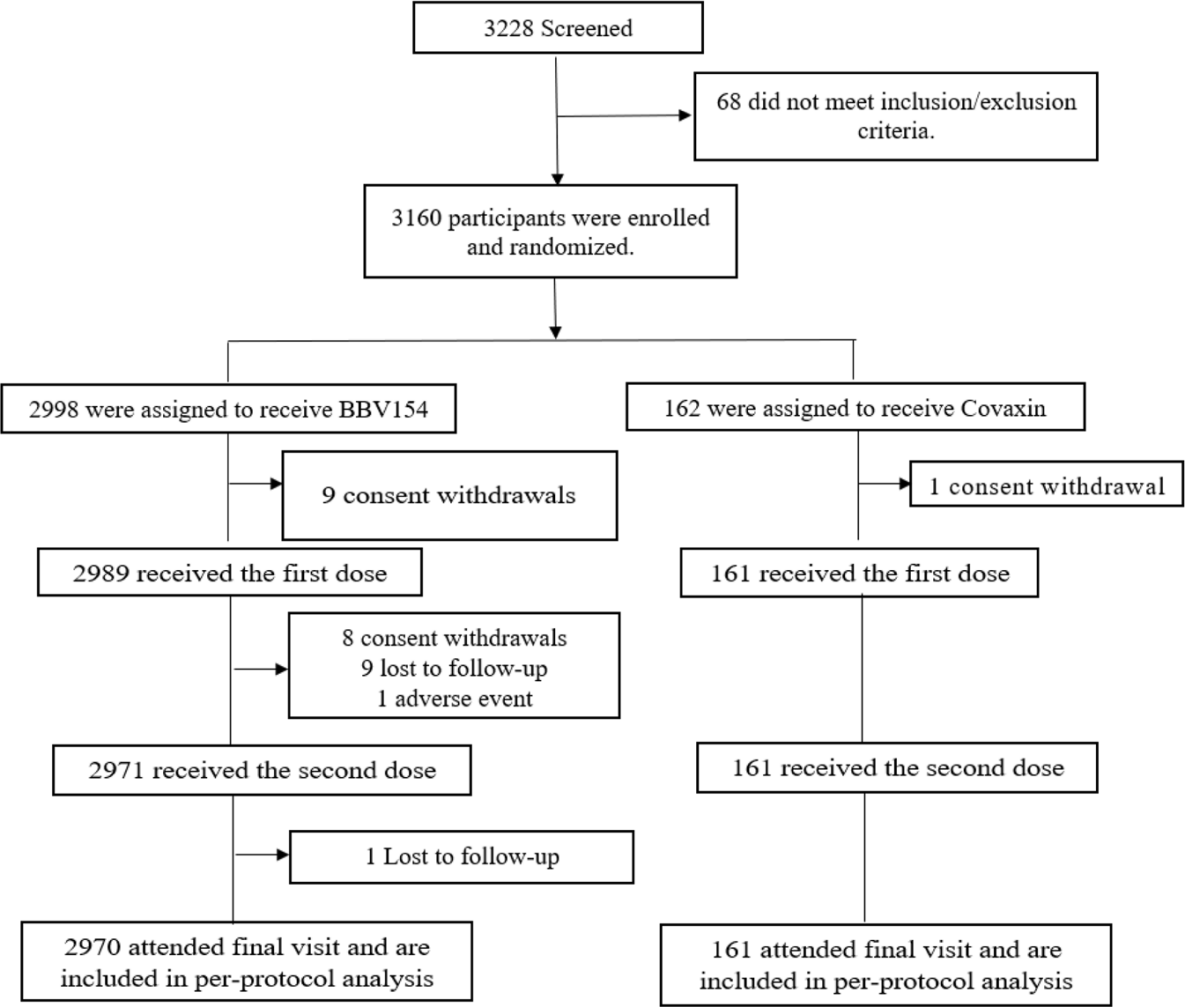
Study flow diagram.

Between April 16 and June 4, 2022 we screened 3228 volunteers, of whom 3160 were enrolled; 2998 participants were randomised to receive BBV154 and 162 to receive Covaxin (Fig. 1). The retention rates at day 42 were 99.1% (2971/2998) and 99.4% (161/162) in BBV154 and Covaxin groups, respectively. There were no meaningful differences in demographic characteristics between the two groups (Table 1).

**Table 1:** Demographic characteristics of the participants in the Intention-to-Treat population.

Parameter	Statistic	BBV154 (*N* = 2989)	Covaxin (*N* = 162)
Age (years)	Mean ± SD	35.9 ± 10.8	34.7 ± 11.2
	Median (Q1; Q3)	34.4 (27.4; 42.5)	33.7 (25.6; 41.7)
	Range	18.0, 80.4	18.2, 78.7
Gender			
Female	*n* (%)	923 (30.8)	46 (28.4)
Male	*n* (%)	2075 (69.2)	116 (71.6)
Height (cm)	Mean ± SD	165.4 ± 7.4	166.2 ± 6.7
	Median (Q1; Q3)	166 (160; 170)	167 (162; 170)
	Range	141, 190	149, 182
Weight (kg)	Mean ± SD	63.96 ± 9.54	65.14 ± 8.93
	Median (Q1; Q3)	64.0 (57.0; 70.0)	65.0 (58.3; 70.4)
	Range	36.7, 108.0	47.6, 92.0
BMI^a^ (kg/m^**2**^**)**	Mean ± SD	23.31 ± 2.70	23.54 ± 2.56
	Median (Q1; Q3)	23.03 (21.48; 24.91)	23.27 (21.97; 24.98)
	Range	16.79, 34.72	18.83, 31.14

^a^BMI: body mass index is the weight in kilograms divided by the square of the height in metres. The calculation was based on the weight and height measured at the time of screening.

### Immunogenicity

For the primary immunogenicity objective, the GMT of neutralising antibodies against wild-type virus at Day 42 in the BBV154 group (all lots) was 768.5 (95% CI: 665.1–888.0) compared with 531 (425.9–662.1) in the Covaxin group; a GMT ratio of 1.45 (95% CI: 1.11–1.88). As the lower bound of the 95% CI was >1, this humoral response to BBV154 was superior to Covaxin. The responses represented 29.4- and 14.4-fold increases over baseline after BBV154 and Covaxin, respectively. Similarly, seronegative subjects showed proportionally increased titers at Day 42, with GMTs 471.1 and 340.6 in the BBV154 and Covaxin group, which resulted in 5234 and 3784 higher fold over the baseline titers, respectively (Table 2).

**Table 2:** SARS-CoV-2 neutralizing antibody titers (PRNT_50_ assay) against ancestral (Wuhan) virus at Days 0 and 42, and Omicron sub-lineages BA.5 at Days 0 and 42, two weeks after the second vaccination with intranasal BBV154 or intramuscular Covaxin.

						GMT ratio (BBV154:Covaxin)	
	Vaccine	*n*	GMT	(95% CI)	GMFR	Ratio	(95% CI) *p* value
**Ancestral (Wuhan) virus – whole population**							
Day 0	BBV154	481	26.1	(18.7‒36.6)	–	0.7	(0.04‒1.37) *p* = 0.30
	Covaxin	159	37.0	(20.9‒65.4)	–		
Day 42	BBV154	481	768.5	(665.1‒888.0)	29.4	1.5	(1.11‒1.88) *p* = 0.006
	Covaxin	159	531.0	(425.9‒662.1)	14.4		
**Ancestral (Wuhan) virus – seronegative at Day 0**							
Day 0	BBV154	138	0.09	(0.09‒0.09)			
	Covaxin	39	0.09	(0.09‒0.16)			
Day 42	BBV154	138	471.1	(300.0‒739.7)	5234	1.4	(0.61–3.12) *p* = 0.43
	Covaxin	39	340.6	(171.5‒676.2)	3784		
**Omicron BA.5**							
Day 0	BBV154	159	10.2	(6.1‒17.1)		2.4	(0.72–8.06) *p* = 0.15
	Covaxin	50	4.2	(1.4‒12.6)			
Day 42	BBV154	161	170.8	(137‒213)	16.7	2.1	(1.18–3.64)
	Covaxin	50	82.4	(48.9‒139)	19.6		*p* = 0.02

Neutralizing responses expressed as PRNT_50_ (reciprocal of dilution achieving 50% neutralization).

Superiority was concluded if either the lower limit of the two-sided 95% CI for the ratio of GMTs (BBV154 GMT: Covaxin® GMT) was ≥1.0.

*GMFR* geometric mean-fold rise from baseline to Day 42.

When measured per lot of BBV154, there were no significant differences between the GMTs of the three lots, at Day 42: 758.3 (95% CI: 591.4–972.3), 741.2 (95% CI: 567.4–968.2), 806.9 (95% CI: 634.6–1026) (see Supplementary Table 2).

Neutralizing responses against Omicron BA.5 was performed with sub-set samples randomly selected from five different sites representing different geographical regions in 3:1 ratio of BBV154 (*n* = 150) and Covaxin (*n* = 50) groups. Neutralising GMTs against Omicron sub-lineage BA.5 at Day 42 were significantly higher (*p* = *0.02*) in the BBV154 group 170.8 (95% CI: 137–213) compared with 82.4 (95% CI: 48.9–139) in the Covaxin group (Table 2). However, both the vaccines showed ~5 fold reduction in neutralization activity; 4.5 fold in BBV154 and 6.4 fold in Covaxin, against Omicron BA.5 variant.

There were high levels of serum IgG antibodies against S-protein at baseline (Supplementary Table 3), which approximately doubled by Day 42 with GMTs of 7175 EU/mL (95% CI: 6490–7932) after BBV154 and 5689 EU/mL (95% CI: 4952–6537) after Covaxin vaccination; a GMT ratio for BBV154 vs Covaxin of 1.3 (95% CI: 1.0–1.5). Both vaccines also showed increased serum IgA antibodies against S-protein, achieving similar GMTs by Day 42: 3069 EU/mL (95% CI: 2794–3371) and 3537 EU/mL (95% CI: 3102–4035) (Supplementary Table 3). There was detectable salivary IgA measured by ELISA at baseline, 10.7 EU/mL (95% CI: 8.4–13.5), and a small increase to 12.3 EU/mL (95% CI: 8.7–17.4) at Day 42 in the BBV154 group. Conversely, in the Covaxin group, there was a decrease from Day 0 to Day 42, from 8.0 EU/mL (95% CI: 5.4–11.8) to 6.6 EU/mL (95% CI: 4.6–9.5).

GMTs of ancestral S-protein specific Th1-dependent IgG1 binding antibodies measured by ELISA and expressed in arbitrary units at Days 0 and 42 were 1008 (95% CI: 301–3376) and 17,688 (95% CI: 8651–36168) in the BBV154 group, and 1270 (95% CI: 359–4492) and 23,702 (95% CI: 13,326–421,567) in the Covaxin group. GMTs of ancestral S-protein specific Th2-dependent IgG4 binding antibodies at Days 0 and 42 were 90.6 (95% CI: 63.9–128) and 190 (95% CI: 106–342) in the BBV154 group and 114 (95% CI: 69.4–186) and 151 (95% CI: 90.9–250) in the Covaxin group. (Supplementary Fig. 1A).

**Fig. 2:**
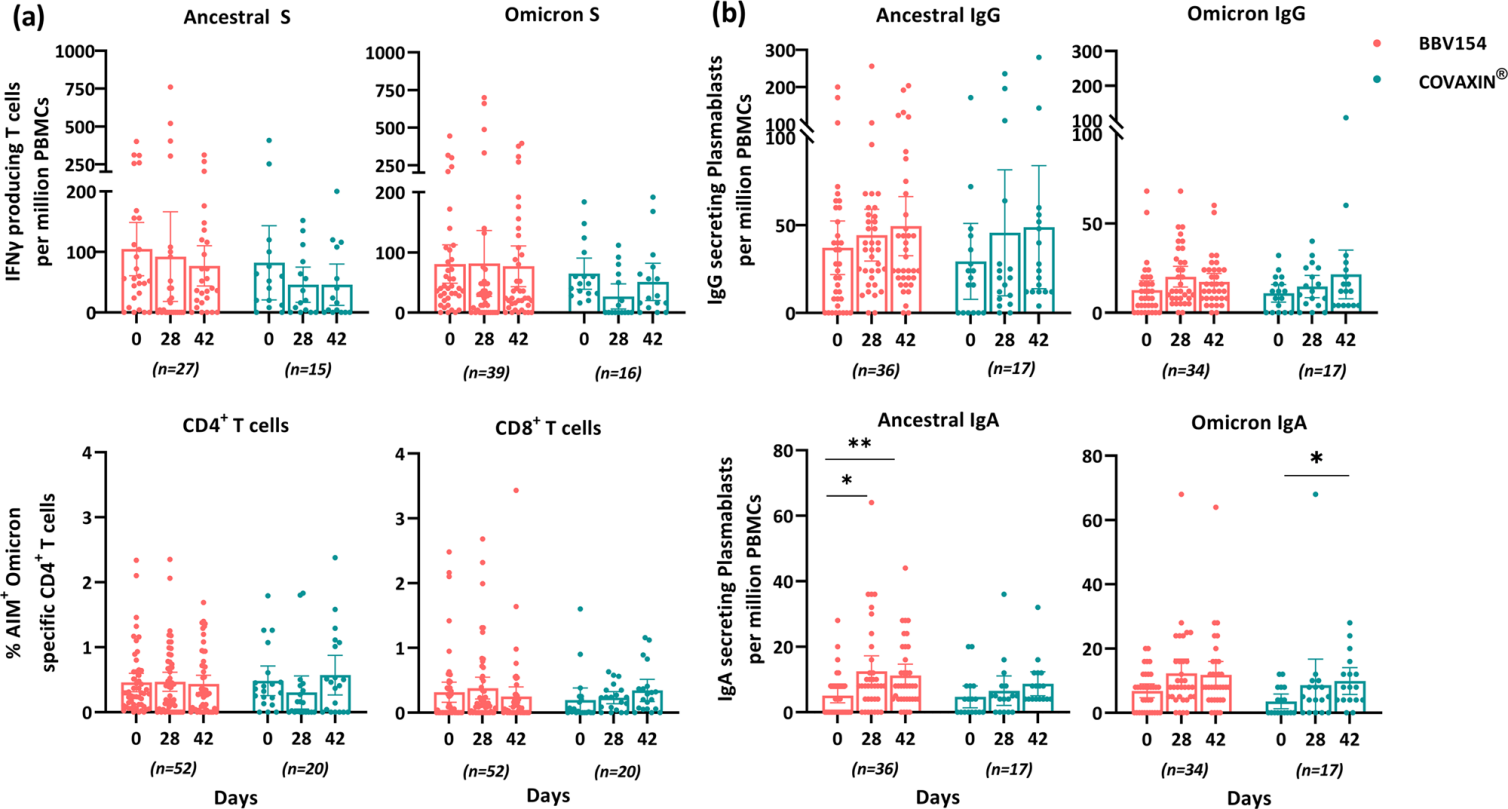
T and B cell responses. PBMCs from vaccinated subjects on Day 0, 28, and 42 were stimulated overnight with either ancestral whole virion inactivated antigen or Omicron strain Spike (S) protein. Unstimulated cells were used as negative controls. **a** SARS-CoV-2-specific IFNγ release was evaluated using ELISPOT (Top two images). SARS-CoV-2 recall responses were demonstrated by the presence of AIM^+^ omicron specific CD4^+^ or CD8^+^ T cells (Bottom two images). Statistical analysis was done by ANOVA (repeated measures) followed by Tukey comparison test to compare the CMI responses observed in BBV154 or Covaxin group at day 28 or 42, versus Day 0. There were no significant differences between time points within each group (BBV154 or Covaxin), except, where asterisk/s indicated. Further, CMI responses observed between BBV154 and Covaxin at different time points were compared by Sidak Multiple comparison test. There were no significant differences between BBV154 vs Covaxin at all time points. **b** Antigen-specific (ancestral and omicron) antibody (IgG and IgA) secreting plasmablasts performed by ELISpot assay. There was statistically significant B cell responses observed in BBV154, after single (Day 0 versus Day 28, *p* < 0.05) and two doses (Day 0 vs Day 42, *p* < 0.01), interms of ancestral specific IgA secreting plasmablasts, but not against Omicron. However, Covaxin induced significant omicron specific IgA secreting plasmablasts at day 42, when compared to Day 0 (*p* < 0.05). Similarly, antigen specific IgG secreting plasmablasts against wuhan or Omicron did not show significant difference between all time points. Further, there was no statistically significant difference between BBV154 and Covaxin across all time points. For brevity, statistical representation for all combinations has not been shown; *p < 0.05, **p < 0.01. Error bars represent 95% confidence intervals.

Spike-protein-specific IFNγ secreting T cell responses against ancestral and Omicron are similar between BBV154 and Covaxin groups across all time points (Days 0, 28 and 42) with a high baseline IFNγ secreting T cell responses. However, in seronegative subjects, though there is an increased response on day 28 in both groups, there is no statistical difference due to the low sample size (Supplementary Fig. 1B). Similarly, SARS-CoV-2 recall responses were demonstrated by the presence of AIM^+^ Omicron-specific CD4^+^ or CD8^+^ T-cells in both groups. The proportions of T_CM_ (CCR7^+^CD45RA^−^) and T_EM_ (CCR7^−^CD45RA^−^) phenotype distribution were high in CD4^+^ T cell populations, and the CD4^+^ CCR7^−^ CD45RA^+^ (T_EMRA_) phenotype was high in CD8^+^ T cell populations (Fig. 2a and Supplementary Figs. 2 and 3)_._ Further, there was no substantial increase in Omicron-specific CD4 or CD8 populations at all time points and between the groups. However, on day 28, there was a slight increase in T_EM_/T_CM_ phenotype within the CD4^+^ T-cell subset and T_EMRA_ phenotype within the CD8^+^ T-cell subset, compared to day 0 in both groups. Perhaps, this increase in CD4^+^ T_EM_/T_CM_ or CD8^+^ T_EMRA_ phenotype could be attributed to the functional activity of the vaccine (Supplementary Fig. 3). Interestingly, the increase was statistically significant in the T_CM_ phenotype (*p* = 0.026) of the BBV154 group.

Antibody-secreting cells (ASCs) induced higher IgG/IgA against both ancestral and Omicron antigens on Day 28 or 42, compared to Day 0. in both BBV154 and Covaxin groups. However, there was a statistically significant increase of IgA-producing plasmablasts against ancestral antigen observed in the BBV154 group (Fig. 2b) on day 28 or 42 compared to day 0, but there is no difference across time points in the COVAXIN group. Further, PBMCs with Omicron stimulation, there is a significant difference in the frequency of plasmablasts on day 42 compared to day 0 in Covaxin group. Overall, BBV154 showed increase inthe frequency of IgA ASCs after single and two doses against ancestral, whereas, Covaxin recipients showed higher frequency of IgA ASCs after two doses. Further, persistence of long-lived memory B cells (MBCs) was demonstrated by the detectable levels of SARS-CoV-2 specific IgG and IgA secreting B cells per million PBMCs from both vaccine (BBV154 and Covaxin) groups (Supplementary Fig. 4).

Vector specific immunity was observed by the low titers of ChAd36 neutralizing antibodies following repeated doses of BBV154 vaccination. However, the immune sera derived from the same recipients displayed significantly higher levels of SARS-CoV-2 virus neutralization activity compared with pre-immune sera (Supplementary Fig. 5)

### Safety and reactogenicity

Up to the interim cut-off point (Day 90), no deaths, hospitalisations, serious adverse events or symptomatic SARS-CoV-2 infections had been reported to the site investigators, with surveillance through telephone follow-up or site visits. However, illness visits were not scheduled, and routine SARS-CoV-2 nucleic acid testing was not conducted. Long-term safety outcomes are still being assessed.

After both doses, the proportions of participants reporting solicited adverse reactions were 6.9% (205/2989) in the BBV154 group and 25.5% (41/161) in the Covaxin group (Table 3). The difference was due to injection site reactions, mainly pain, in the Covaxin group. The most frequently reported being rhinorrhoea in 2. 7% (78/2989) of the BBV154 group, and pain at injection site in 16.8% (27/161) of the Covaxin group. All systemic adverse events were described as mild and transient and resolved within the 24 h. A total of 50 unsolicited adverse events were reported, 45 (1.2%) in BBV154 and 5 (3.1%) in Covaxin group. No significant difference was observed between the groups (Table 4).

**Table 3:** Solicited local and systemic adverse events reported within 7 days after administration of BBV154 or Covaxin.

	[*m* events] in *n* participants (%)	
Adverse events	BBV154 (*N* = 2989)	Covaxin® (*N* = 161)
Overall	[240] 205 (6.9%)	[51] 41 (25.5%)
Injection site pain	[0] 0 (0.0%)	[30] 27 (16.8%)
Injection site erythema	[0] 0 (0.0%)	[5] 5 (3.1%)
Injection site swelling	[0] 0 (0.0%)	[6] 6 (3.7%)
Lacrimation increased	[1] 1 (0.0%)	[0] 0 (0.0%)
Nasal congestion	[6] 6 (0.2%)	[0] 0 (0.0%)
Oropharyngeal pain	[16] 16 (0.5%)	[0] 0 (0.0%)
Rhinalgia	[15] 15 (0.5%)	[0] 0 (0.0%)
Rhinorrhoea	[79] 78 (2.67%)	[0] 0 (0.0%)
Sneezing	[42] 41 (1.4%)	[0] 0 (0.0%)
Chills	[1] 1 (0.0%)	[0] 0 (0.0%)
Fatigue	[4] 4 (0.1%)	[0] 0 (0.0%)
Headache	[47] 44 (1.5%)	[1] 1 (0.6%)
Myalgia	[7] 7 (0.2%)	[0] 0 (0.0%)
Nausea	[2] 2 (0.1%)	[2] 2 (1.2%)
Pyrexia	[17] 18 (0.6%)	[6] 5 (3.1%)
Vomiting	[2] 2 (0.1%)	[1] 1 (0.6%)

*m* = No. of adverse events.

*n* = No. of participants and % is percentage of participants with the adverse event in respective treatment group.

**Table 4:** UnSolicited adverse events reported after administration of BBV154 or Covaxin.

Adverse events	BBV154 (*N* = 2989) [*m*] *n* (%)	Covaxin® (*N* = 161) [*m*] *n* (%)
Overall	[45] 36(1.2%)	[5] 5(3.1%)
Cough	[13] 13 (0.8%)	[2] 2 (1.2%)
Rhinorrhoea	[3] 3 (0.1%)	[2] 2 (1.2%)
Nasal pruritus	[4] 3 (0.1%)	[0] 0 (0.0%)
Nasal congestion	[2] 2 (0.1%)	[0] 0 (0.0%)
Nasal discomfort	[1] 1 (0.0%)	[0] 0 (0.0%)
Sneezing	[1] 1 (0.0%)	[0] 0 (0.0%)
Lower respiratory tract infection	[1] 1 (0.0%)	[0] 0 (0.0%)
Upper respiratory tract infection	[1] 1 (0.0%)	[0] 0 (0.0%)
Pyrexia	[3] 3 (0.1%)	[0] 0 (0.0%)
Fatigue	[2] 2 (0.1%)	[0] 0 (0.0%)
Malaise	[0] 0 (0.0%)	[2] 2 (1.2%)
Chills	[1] 1 (0.0%)	[0] 0 (0.0%)
Headache	[5] 5 (0.2%)	[0] 0 (0.0%)
Dizziness	[1] 1 (0.0%)	[0] 0 (0.0%)
Diarrhoea	[1] 1 (0.0%)	[1] 1 (0.6%)
Stomatitis	[1] 1 (0.0%)	[0] 0 (0.0%)
Irritability	[2] 2 (0.1%)	[0] 0 (0.0%)
Radius fracture	[1] 1 (0.0%)	[0] 0 (0.0%)
Rhinitis	[1] 1 (0.0%)	[0] 0 (0.0%)
Rhinorrhoea	[1] 1 (0.0%)	[0] 0 (0.0%)
Vestibular neuronitis	[1] 1 (0.0%)	[0] 0 (0.0%)
Myalgia	[1] 1 (0.0%)	[0] 0 (0.0%)

*m* = No. of adverse events, *n* = No. of participants.

% is calculated by dividing the No. of participants reporting AE by total No. of participants in respective treatment group.

## Discussion

In this report of the interim findings from a phase 3 clinical trial, we found that two weeks after a second vaccination with BBV154, an intranasal, adenoviral-vectored SARS-CoV-2 vaccine, neutralization titres against wild-type (D614G) SARS-CoV-2 virus were superior to those observed two weeks after two doses of the intramuscular Covaxin vaccine. Similarly, the intranasal vaccine induced significantly higher cross-neutralizing responses against the BA.5 sub-lineage of the Omicron variant. In addition to these humoral responses, we also detected higher mucosal (sIgA) antibodies at Day 42 following BBV154 compared with Covaxin administration. These results were further supported by statistically significant increases of IgA-secreting plasmablasts on Day 42, compared with Day 0 in the BBV154 group. Both vaccines induced IgG1-mediated responses. Further evaluation of extensive T cell mediated immunity studies revealed high baseline T cell responses in both groups. Presumably, this could be due to prior infection and which probably masked the vaccine-induced T cell responses in both groups and the T cell responses were comparable in both groups. Further analysis of CMI data in seronegative vs seropositive subjects could not be done due to the limited sample size. Both BBV154 and Covaxin were generally well tolerated with very low reactogenicity rates and no reported vaccine-related serious adverse events.

In this study, the combined incidence rates of local and systemic adverse events after the first and second doses of BBV154 are strikingly lower than the rates reported for other SARS-CoV-2 vaccine platform candidates^14–19^. However, other vaccine studies enrolled different populations and employed varying approaches to measure adverse events. Nonetheless, the intranasal route of administration was well tolerated in comparison with the injected Covaxin control vaccine, with fewer than 5% and 3% of vaccinees reporting local or systemic adverse events.

Mucosal immunisation potentially provides several advantages over conventional intramuscular vaccination, mostly against respiratory diseases. Mucosal (secretory) IgA plays a crucial role in protecting mucosal surfaces against pathogenic respiratory viruses by blocking their attachment to epithelial cells. Influenza-specific IgA has been shown to be more effective in preventing infections in mice and humans than influenza-specific IgG, and elevated IgA serum levels correlate with influenza vaccine efficacy^20^. SARS-CoV-2 initially infects the upper respiratory tract, and a rapid rise in plasma IgA antibodies that bind to SARS-CoV-2 persists for at least 30–40 days^21^. This suggests that IgA-mediated mucosal immunity may be a critical defence mechanism against SARS-CoV-2. Further, it has been shown that IgA dimers, the primary form of antibody in the nasopharynx, were an average of 15 times more potent than IgA monomers against the same target. Thus, secretory (dimeric) IgA responses may be particularly valuable for protection against SARS-CoV-2 and for vaccine efficacy^22^. Preclinical studies have demonstrated that intranasal inoculation but not intramuscular injection of influenza vaccine induces potent local antigen-specific T cell responses in the lungs that are crucial for sterilizing immunity^23^. Further, one intranasal inoculation of mice with dNS1-RBD elicited a robust T-cell immune response in lung tissue about 26 times stronger than in PBMCs^24^. Preclinical studies showed that intranasal inoculation with BBV154 could induce SARS-CoV-2-specific CD8^+^ T cells in the lung, including CD103^+^ CD69^+^ cells, which are likely of a resident lineage (TRM). Lung TRM cells can provide stronger protective immunity than circulating T cells^10^. However, it is difficult to analyse immune response in the lungs in clinical trials as human lung sampling is unrealistic, which might lead to an underestimation of the intensity of cellular immunity of intranasal vaccines clinically.

Both vaccines showed detectable Omicron-specific (ancestral and Omicron) IFN-γ producing T cells and the responses are comparable, with a majority of CD4^+^ T_CM_ and CD4^+^ T_EM_, including distinct CD8^+^ T_EMRA_ phenotype, demonstrating a durable and persisting immune T cell memory response. Distinct CD8^+^ T_EMRA_ phenotype induced by both vaccines could be attributed to the cytotoxic function of CD8 T cells upon antigen exposure. These T-cell responses were comparable in both groups. BBV154 also induced significant levels of IgA secreting plasmablasts, which correlated with an increase in its neutralization potency against both homologous and heterologous strains.

Intranasal vaccines offer several potential advantages over parenteral immunisation, including ease of administration, non-invasiveness, improved patient compliance, and suitability for mass vaccination. Adenoviral vectors are already used in a variety of vaccines, both licensed or in development, including against COVID-19, Ebola, and tuberculosis, and can be scaled to meet the global vaccine demand with fewer side effects and ease of production and low cost. Furthermore, robustness and balanced immune responses induced by the adenoviral-vectored vaccines made them an effective approach to counter the COVID-19 pandemic^25^.

The immunity against adenovirus capsid proteins can reduce the efficacy of adenoviral-vectored vaccines, particularly in parenteral administration. To compensate for the pre-existing immunity, a relatively high dosage of Ad5 vectored Ebola virus vaccine was used in seropositive individuals^26,27^. In contrast, intranasal delivery of recombinant (human and chimpanzee) adenovirus-based vaccines has been shown to circumvent pre-existing immunity and confer sufficient protection against challenge with a variety of pathogens^28^. Consequently, the absence or insignificant levels of ChAd36 neutralizing antibodies following repeated doses of BBV154 vaccination in both preclinical^11^ and this clinical study (Supplementary Fig. 5) implies that intranasal administration may offer an advantage even after repeated vaccination of adenovirus vectored vaccines.

This study had several strengths, notably being conducted in diverse geographic locations within India to ensure generalisability, but it also has several limitations. Recruitment of participants was based on the oral declaration of the volunteers that they had no history of symptomatic infections. However, the high baseline titers in both groups indicates that there could be a possibility of asymptomatic infection. Further, this study was conducted at a time when the third Omicron wave was occurring in India^29^. However, the effectiveness of BBV154 in seronegative subjects showed a remarkable increase in neutralization titers compared to Covaxin, in terms of fold increase, suggesting that BBV154 is an effective primary series vaccine in naïve subjects.

Similarly, prior infections seem to have impacted vaccine-induced mucosal responses, as the magnitude of saliva IgA response in BBV154 vaccinees was only about two-fold higher than in Covaxin recipients. However, in two Phase 2 clinical studies (CTRI/2021/09/036257 & CTRI/2021/08/035993) that we conducted during August– September 2021, when the infection rate was much lower, BBV154 induced 4 to 5-fold higher saliva IgA titers compared with baseline in seronegative subjects (*see* Supplementary Table 4). Possibly, these results would have implications on the ability of BBV154 to block infection and forward transmission of SARS-CoV-2 in the community, depending on the severity of the infection. Although we did not observe any breakthrough COVID-19 cases in either of the groups, illness visits were not scheduled, and routine SARS-CoV-2 nucleic acid testing was not conducted. Therefore, the results reported here do not permit efficacy assessments, which require large-scale studies to evaluate the reduction in the severity of breakthrough infections and transmission.

The present findings demonstrate the superiority of the humoral immune response to BBV154 compared with Covaxin, and as the latter has proven efficacy against SARS-CoV-2 variants^30^, we may speculate that BBV154 is also efficacious. However, the peak of the COVID-19 pandemic due to the ancestral (Wuhan) SARS-CoV-2 has passed, and ongoing outbreaks and new waves of disease are due to emerging variants. We have shown that BBV154 also elicits immune responses against one of the most recently emerged variants, Omicron sub-lineage BA.5, which suggests that there will likely be some efficacy against SARS-CoV-2, but this remains to be determined. Accordingly, analysis of neutralization antibody titers also revealed that both vaccine groups did show neutralization effectiveness against Omicron BA.5, though, there was fourfold reduction in titers compared to Nab tires against ancestral (Wuhan). However, more than 80% of the BBV154 vaccinees did respond and showed titers 10-fold higher than the baseline titers.

Evaluation of safety outcomes and Vaccine-Induced Thrombotic Thrombocytopenia (VITT) will require larger studies, and ongoing follow-up on studies (planned in this study with post-vaccination visits at months 6 and 12) will be required to establish the durability of the immune responses as well as immunogenicity and tolerability in children and the elderly.

Two other mucosal SARS-CoV-2 vaccines have shown mixed results in clinical development. In a phase 1 trial, two doses of an intranasally administered ChAdOx1 nCoV-19 failed to show immunological (mucosal and systemic) equivalence when compared with an injected ChAdOx1 nCoV-19^18^. Although further study is warranted, we note several differences with the intranasal ChAdOx1 nCoV-19 trial, including proline stabilization of the spike antigen, vector changes that could impact spike protein expression, distinct modes of vaccine administration (nasal drops versus inhalation), different ChAd serotypes that could impact vaccine tropism for cells in the upper respiratory tract, and a higher vaccine dose volume of 0.5 mL for BBV154. A heterologous booster dose of an aerosolised adenovirus type-5 vectored COVID-19 vaccine (Ad5-nCoV) showed immunological superiority with a homologous injectable inactivated booster^19^.

In conclusion, we have demonstrated that the intranasal application of two doses of BBV154 was generally well tolerated by adults, with none of the pain associated when compared with the intramuscular injections of Covaxin (control vaccine) and a lower rate of systemic adverse events, while eliciting a superior humoral neutralising response against the ancestral strain SARS-CoV-2 virus, and cross-neutralising activity against an Omicron variant. Further clinical development of BBV154 is ongoing, including as part of a heterologous booster regimen in phase 3 clinical trials (Clinical Trials.gov, identifiers NCT05567471).

## Methods

### Trial design and participants

The study was a randomised, open-label, multicentre trial to evaluate the immunogenicity and safety of BBV154 in healthy adults across 14 hospitals in India. The study protocol was approved by the National Regulatory Authority (India) and the respective Ethics Committees at each hospital centre (see Supplementary Table 1). It was registered on the Indian Clinical Trials Registry India, CTRI/2022/02/40065, and ClinicalTrials.gov: identifier, NCT05522335. The trial was conducted in compliance with all International Council for Harmonisation (ICH) Good Clinical Practice guidelines. The objective was to assess and compare the immunogenicity and tolerability of the intranasal vaccine with the licensed intramuscular vaccine, Covaxin.

Eligible participants were healthy males or non-pregnant females aged ≥18 years, at the time of enrolment who had never previously received any COVID-19 vaccine and had no reported history of SARS-CoV-2 infection. Proof of non-vaccine status was confirmed by consulting the Indian electronic COVID-19 database (CoWIN). The main exclusion criteria were being pregnant or breastfeeding in women, or having any co-morbidities. All participants were screened for eligibility based on their health status, including their medical history, vital signs, and physical examination. They were enrolled after providing signed and dated informed consent forms after having all required study-related activities and the availability to decline or withdraw from the study explained to them.

### Study vaccines

BBV154 (Bharat Biotech, Hyderabad, India) is a chimpanzee adenoviral-vectored SARS-CoV-2 intranasal vaccine encoding a prefusion stabilised spike (S) protein based on the ancestral (Wuhan) strain. Three consecutive manufacturing lots were used to obtain lot-to-lot consistency data for regulatory assessment. The control vaccine, Covaxin® (BBV152, Bharat Biotech, Hyderabad, India), is a whole-virion ß-propiolactone-inactivated SARS-CoV-2 vaccine. Each dose 0.5 mL contains 6 μg of antigen formulated with the toll-like receptor 7/8 agonist molecule, imidazoquinoline gallamide (IMDG), chemisorbed onto aluminium hydroxide.

### Outcomes

For the primary outcome, the humoral neutralising antibody titer was measured by plaque-reduction neutralization test (PRNT_50_) using wild-type virus (D614G) at Bharat Biotech. Secondary immunogenicity outcomes were humoral and mucosal IgA titers measured by enzyme-linked immunosorbent assay (ELISA), and cell-mediated responses. The lot-to-lot consistency requirement was based on neutralising titers at Days 0 and 42. Secondary tolerability outcomes were the numbers and percentages of participants with solicited local reactions within two hours, systemic adverse events within 7 days and unsolicited adverse events within 28 days of either vaccination. Cross-protection against Omicron variants were evaluated as an exploratory outcome.

### Randomisation and masking

A total of 3160 subjects were enrolled and randomized in the study. Of which 2520 population considered for safety and 640 considered for immunogenicity and safety in the ratio of 3:1 (BBV154 : Covaxin) with a block size of 4. Three batches of BBV154 were used for lot to lot comparision in equal distribution (*n* = 160 per batch).

Master randomisation lists with randomisation number and intended allocation were prepared by a contract research organisation (George Clinical).

### Statistical analysis

A total of 3000 study participants were enrolled in the BBV154 study arm and 160 in the Covaxin® arm. The first 480 BBV154 recipients and all 160 Covaxin® recipients were randomised in blocks of size divisible by 4. Within each block, vaccine assignments were in the ratio of 3:1 (BBV154: Covaxin®). Assuming a 5% loss in each group due to withdrawal, loss to follow-up, etc., would leave approximately 456 BBV154 recipients and 152 Covaxin vaccinees for analysis. Based on our previous study data of BBV154 and Covaxin®, we assumed a geometric mean titer (GMT) of 700 (mean log_10_ titer = 2.845) for BBV154 and 500 (mean log_10_ titer = 2.699) for Covaxin® and a standard deviation of log_10_ titer of 0.4 for BBV154 and 0.6 for Covaxin® (CTRI/2021/08/035993). In a power determination comparing BBV154 and Covaxin® by a two-sample t-test at the two-sided 5% significance level, allowing for unequal standard deviations, the power to show a significantly higher GMT for BBV154 was ~80%. Superiority was concluded if either the lower limit of the two-sided 95% CI for the ratio of GMTs (BBV154 GMT: Covaxin® GMT) was ≥1.0, or alternatively, the GMT was larger for BBV154 than for Covaxin® and the two-sided *p*-value for a t-test comparing means of log_10_ titer was ≤0.05.

Consistency between the three consecutively manufactured lots of BBV154 vaccine was based on the neutralizing antibody GMT ratios for each pair of lots on Days 0 and 42, with the corresponding two-sided 95% CIs calculated from CIs for log_10_-transformed neutralising antibody titer, using t-distributions. The criterion for lot consistency was that two-sided 95% CIs for GMT ratios for all pairs of lots must be contained within the interval [0.5, 2.0]. GMTs with 95% confidence intervals (CIs) were presented for immunological endpoints. For continuous variables (below 20 observations), medians and IQRs (Interquartile range) were reported. The exact binomial calculation was used for the CI estimation of proportions. CI estimation for the GMT was based on the log_10_ (titer) and the assumption that the log_10_ (titer) was normally distributed. A comparison of GMTs was performed by *t*-tests on the means of the log_10_ (titer). Significance was set at *p* < 0.05 (two-sided).

Safety analyses included all participants who were vaccinated and provided any safety data on immediate AEs within 30 min of vaccination, solicited local or systemic AEs within 7 days after each dose of vaccine or any AEs and SAEs throughout the study (up to Day 90). These categories of events were compared between treatment groups using two-sided z-tests or Fisher exact test. Safety endpoints were described as frequencies (%). Descriptive and inferential statistics were performed using SAS 9.4.

### Procedures

Following a baseline blood draw on Day 0, the first dose of either vaccine was administered, and a second dose was given on Day 28. Each dose of BBV154 was formulated as 0.5 mL in single dose vials. No on-site dose preparation was required. A sterile/disposable dropper was installed onto the opened vial, the participant was instructed to lie down with head slightly tilted back with chin facing the ceiling, and four drops (0.25 mL) of BBV154 were administered into each nostril. The participant was asked to remain prone for 30 s. Covaxin was administered by intramuscular injection in the deltoid. Participants were observed for 30 min post-vaccination to assess immediate reactogenicity. They then completed paper diary cards which solicited local reactions and systemic adverse events daily for seven days after each vaccination, including time of symptom onset, severity, time to resolution, and concomitant medication. Routine telephone calls were scheduled during the first seven days to ensure dairy card completion, and cards were collected at the next visit to the site. Solicited local reactions to BBV154 included nasal congestion, cold, dryness, pain, sneezing, and to Covaxin were injection site pain, erythema and swelling. Systemic adverse events for both vaccines included fever, fatigue/malaise, myalgia, body aches, headache, nausea/vomiting, anorexia, chills, generalized rash, and diarrhoea. Vaccine-induced thrombotic thrombocytopenia (VITT) was evaluated in all participants within 28 days of receiving any vaccination. Participants recorded any unsolicited adverse events for 28 days after each vaccination (Table 4), which were graded according to severity score (mild, moderate, or severe) and whether, in the investigator’s opinion, they were related or not related to the investigational vaccine.

Blood was drawn from all sites for immunogenicity assessments on Days 0, 28 and 42. However, Subsets of participants from three sites (20 from each lot group in the BBV154 arm and 20 from the Covaxin arm) who consented to provide additional 10 mL whole blood samples for collection of peripheral blood mononuclear cells (PBMC), and 5 mL saliva sample was collected from one site, on Days 0, 28, and 42 for the measurement of salivary IgA (sIgA). Sites for the saliva and PBMC sample collection were chosen selectively, inorder to improve the quality of sample and to reduce the time between samples collection and their process.

### Immunogenicity assessments

Immunogenicity analyses were done in a blinded manner. Neutralising antibody titers against ancestral SARS-CoV-2 (Wuhan) and the Omicron BA.5 sub-lineage were determined by a plaque-reduction neutralization test with titers expressed as the reciprocal of the dilution that achieved a 50% reduction in virus (PRNT_50_). Humoral SARS-CoV-2 spike-specific IgG and IgA (serum) responses and sIgA in saliva against the ancestral SARS-CoV-2 spike (S1) protein were measured by enzyme-linked immunosorbent assay (ELISA) and expressed as geometric mean titers (GMTs) in ELISA units per mL (EU/mL)^30^. SARS-CoV-2 spike-specific immunoglobulin subclasses (IgG1 and IgG4, respectively) were assessed by ELISA on Days 0 and 42 and expressed as GMTs^30^. PBMC collected on days 0, 28, and 42 were used for cell-mediated immunity (both T and B cells) by ELISpot and AIM (activation-induced marker) assays at Immunitas Biosciences (Bangalore, India). Other assays were performed at Bharat Biotech.

### Enzyme-linked immunosorbent assay (ELISA)

SARS-CoV-2 specific antibody binding titers either IgG or IgA from both serum and Saliva were determined by ELISA. The main principle of ELISA is the same across all the assays; Briefly, microtiter plates were coated with a spike (S1) protein (Syngene, Bangalore, India, Batch No# PRB033635), at a concentration of 1 µg/ml, 100 µl/well in PBS pH 7.4. After overnight incubation, wells were blocked and serially diluted sera or saliva samples were added. After incubation, appropriate secondary antibody dilution was added followed by the addition of Tetramethyl benzidine as a substrate. Absorbance was measured at 450/630 nm. Known positive and negative controls from the previous phase 1 and 2 adult clinical trials was also maintained as an assay control. The threshold value (Mean + 3 SD) was established by taking the absorbance of negative control samples. The reciprocal of the antibody dilution at which absorbance is above the threshold was taken as the antigen-specific antibody endpoint titers, for sera samples collected on Days 28 and 42. Detailed description for each assay is as follows.

To assess the SARS-CoV2 spike (S1) IgG binding antibody titers, serum was diluted serially (2fold) starting from 1:200 to 51,200 (if endpoint titer was found to be more than 51,200, samples were retested at higher dilutions, >51,200) and added to pre-coated plate. After incubation, Goat anti-Human IgG HRP conjugate (Sigma-Aldrich, Cat. A8667, dilution 1:10,000) was added to the wells and incubated for 1 hr at RT. Negative samples were assigned a titer of 400, for mean titer calculations.

To assess the SARS-CoV2 spike (S1) IgA binding antibody titers, serum was diluted serially starting from 1:100 to 6400 and added to pre-coated plate. After incubation, Goat anti-Human IgA HRP conjugate (Sigma-Aldrich, Cat. A0295, dilution 1:2500) was added to the wells and incubated for 1 hr at RT. Negative samples were assigned a titer of 200, for mean titer calculations.

To assess the SARS-CoV2 spike (S1) secretory IgA (sIgA) binding antibody titers from Saliva, sera was diluted serially from 1:4 to 1:256 and added to pre-coated plate. After incubation, Goat anti-Human secretory IgA HRP conjugate (Nordic MU Bio, Cat. SH Ahu/Sc/PO, dilution 1:2500) was added to the wells and incubated for 1 h at RT.

To assess SARS-CoV2 spike (S1) antibody (IgG1/IgG4) Isotyping, Immunoglobulin subclasses (IgG1 *vs*. IgG4) were determined by ELISA as described previously^31,32^. Mouse anti Human IgG1 HRP (Cat. A-10648, Invitrogen) or IgG4 (Cat. A-10654, Invitrogen) antibodies at a dilution of 1:1000 were used in the assay.

### Plaque Reduction Neutralisation Test (PRNT50) -

The plaque reduction neutralization test (PRNT) was performed in a biosafety level 3 facility as described previously^31,33^.

### IFN-γ responses by ELISpot Assay

ELISPOT assay was performed using the IFN-γ ELISPOT kit (MABTECH), as per the manufacturer’s instructions. The PBMCs collected on Day 0, 28 and 42 from BBV154 and COVAXIN® groups were used and stimulated with SARS-CoV-2 peptide matrix (peptide pool of SARS-CoV-2 S or S, M & N).

### Activation-induced marker (AIM) assay

Activation Induced marker assay was performed as reported earlier^3^. Briefly, PBMCs (0.7 million cells/100 µl) were plated onto separate 96 well U bottom plates and stimulated with the cocktail of SARS-CoV-2 peptide pool (1 µg/mL). After overnight stimulation, cells were washed and stained 40 minutes with antibody cocktail containing the following fluorescently conjugated antibodies obtained from Biolegend, USA: CD3-APC-A750 (Cat. 300470), CD4-PB450 (Cat. 300521), CD8a-APC-A700 (Cat. 301028), OX40-APC (Cat. 350008), CD137-ECD (Cat. 309826), CD69-PC7 (Cat. 310912), CCR7-PE (Cat. 353204), CD45RA-FITC (Cat. 304148). The plates were centrifuged and the resuspended cells were labelled again with 7AAD solution (Cat. 6604104, Beckman) and analysed in a flow cytometer (CytoFlex S, Beckman Coulter).

### Human IgG/IgA double colour enzymatic ELISpot assay

B-cell ELISpot assay was used to evaluate the frequency of plasmablasts and memory B cells in PBMCs, who received either of the vaccine. The assay has been performed as per the instruction manual and also as previously described^34,35^. As the detection of plasmablast does not require polyclonal stimulation, PBMCs were directly plated onto antigen-coated ELISpot plates. ELISpot plates were coated overnight at 4 °C with S1-protein of omicron and whole inactivated antigen of ancestral virus (100 ng/well).

For memory, B-cell assays, human PBMCs were revived and resuspended in RPMI complete media and stimulated with 1:1000 of polyclonal B (Poly-B) cell activator solution (CTL-hBPOLYS-200, CTL) for 4 days at 37 °C. On Day 4, the plate was blocked and added with PBMC’s (0.3 × 10^6^ cells/well), kept for incubation at 37 °C, with 5% CO_2_ for 16–18 h.

The plate was washed and added diluted detection solution of Anti-human IgA (FITC) and Anti-human IgG (Biotin) and incubated at RT for 2 h. After sufficient washes, the tertiary solution containing FITC-HRP and SA-AP was added and incubated at RT (in dark condition) for 1 h. Spots were developed by the addition of TrueBlue or TrueRed for the visualization of IgG and IgA-secreting cells respectively. Assay controls, unstimulated cells and cells stimulated with Influenza antigen were maintained. Plates are dried and read with the help of an ELISpot reader.

### Reporting summary

Further information on research design is available in the Nature Research Reporting Summary linked to this article.

### Supplementary information

Reporting Summary

Supplementary Material

## Data Availability

BBV154, a chimpanzee adenoviral-vectored SARS-CoV-2 intranasal vaccine encoding a prefusion-stabilized spike protein with two proline substitutions in the S2 subunit (GenBank: QJQ84760.1). The authors also declare that the data supporting the findings of this study are available within the main manuscript or the supplementary material. Correspondence and requests for materials should be addressed to K.M.V. (kmohan@bharatbiotech.com).
