# Supplementary material for: Phase III Pivotal comparative clinical trial of intranasal (iNCOVACC) and intramuscular COVID 19 vaccine (Covaxin®)
Source: NPJ Vaccines. 2023 Aug 18;8:125. doi: 10.1038/s41541-023-00717-8 (PMC10439197; doi:10.1038/s41541-023-00717-8)
Supplement: Supplementary file 2 — Supplementary Material [file 41541_2023_717_MOESM2_ESM.pdf]

Supplementary material for Chandramani Singh et al.

Phase III Pivotal comparative clinical trial of intranasal (iNCOVACC) and intramuscular COVID 19 vaccine (Covaxin®)

Table of Contents

Supplementary data

Table 1: List of clinical sites and Ethical Committees registrations 2

Table 2: Lot-to-lot neutralizing antibody comparisons 3

Table 3: SARS-CoV-2 S1 protein specific IgG and IgA binding antibody responses 4

Table 4: Additional Saliva IgA data 5

Figure S1: Immunoglobulin Subclass Analysis 6

Figure S2: Gating Strategy used to Select SARS-CoV-2 Specific $CD4^+$ (A) & $CD8^+$ (B) T-cell Population 8

Figure S3: Memory T cell phenotype distribution 10

Figure S4: IgG/IgA producing Memory B cells 11

Figure S5: Nasal delivery of BBV154 is less likely to elicit vector specific antibodies 12

This supplementary material has been provided by the authors to give readers additional information about their work.

Supplementary Table 1: List of clinical sites and Ethical Committees registrations

	Site name	Ethics Committee details	EC registration
1	Aatman Hospital, Ahmedabad	Institutional Ethics Committee Aatman Hospital, 5 Anveshan row house, Bhopal, Ahmedabad	ECR/1565/INST/GJ/2021
2	Maharaja Agrasen super specialty Hospital, Jaipur, Rajasthan	Ethics Committee for Research on Human Subjects, Central Spine, Agrasen Aspatal Marg, Sector-7, Vidyadar Nagar, Jaipur, Rajasthan	ECR/1222/INST/RJ/2019/RR-22
3	Redkar Hospital and Research Center, Goa	Redkar Hospital and Research Center Institutional Ethics Committee, Oxelbag, Village-Dhargal, Tal-Pernam, Goa	ECR/902/INST/GA/2018/RR-22
4	Jeevan Rekha Hospital, Belgaum, Karnataka	Institutional Ethics Committee Jeevan Rekha Hospital Dr.B.R Ambedkar Road, Opp Civil Hospital-Belagavi, Karnataka	ECR/1242/INST/KA/2019
5	Pt. BD Sharma Postgraduate Institute of Medical Sciences (PGIMS), Rohtak, Haryana	Institutional ethics committee, Pt. BD Sharma Postgraduate Institute of Medical Sciences (PGIMS), Rohtak	ECR/495/INST/HR/2013/RR-20
6	Rajarajeswari Medical College and Hospital, Bangalore, Karnataka	Institutional Ethics Committee, Kambipura, Mysuru Road, Bangalore	ECR/56/INST/KA/2013/RR-19
7	Visakha Institute of medical science, Visakhapatnam, Andhra Pradesh	Institutional Ethics Committee, Visakha institute of medical sciences, Visakhapatnam	ECR/1421/INST/AP/2020
8	Malla Reddy Narayana Multispeciality Hospital, Hyderabad, Telangana	Institutional Ethics Committee Malla Reddy College for Women, Quthbullapur, Hyderabad.	ECR/981/INST/AP/2017/RR-20
9	Oyster and Pearl Hospitals (Phadnis clinic Pvt.Ltd), Pune, Maharashtra	O & P Institutional ethics committee, Pune	ECR/71/INST/MH/2013/RR-19
10	AIIMS, Patna, Bihar	Institutional ethics committee, AIIMS, Patna	ECR/1387/INST/BR/2020
11	Prakhar Hospital Pvt Ltd. Kanpur, Uttar Pradesh	Ethics committee of Prakhar Hospital, Kanpur	ECR/1017/INST/UP/2017/RR-21
12	Rana Hospital Pvt. Ltd, Gorakhpur, Uttar Pradesh	Institutional ethics committee, Rana Hospital, Gorakhpur	ECR/1332/INST/UP/2020
13	Acharya Vinobha Bhave Rural Hospital, Wardha, Maharashtra	Institutional ethics committee of DMIMS, Wardha	ECR/440/INST/MH/2013/RR-19
14	NIMS, Hyderabad, Telangana	NIMS Institutional ethics committee, Hyderabad	ECR/303/INST/AP/2013/RR-19

Supplementary Table 2: SARS-CoV-2 neutralising antibody titres ($PRNT_{50}$ assay) across three lots of BBV154 for the lot-to-lot comparison

	**Geometric mean titers** (95% CI) **at Day 42 in the three BBV154 study group sub-sets who received different lots of vaccine**		
	**BBV154**		
	**Lot 1**	**Lot 2**	**Lot 3**
N =	160	159	162
**Day 0**	**21·2** (11·4, 39·2)	**35·0** (20·0, 61·3)	**24·2** (13·.5, 43·3)
N =	160	159	162
**Day 42**	**758·3** (591·4, 972·3)	**741·2** (567.4, 968.2)	**806·9** (634.6, 1026)
**GMT Ratio Day 42/Day 0**	**35.8**	**21.2**	**33.3**
	**Lot-to-lot comparisons**		
	**Lot 1 vs. Lot 2**	**Lot 1 vs. Lot 3**	**Lot 2 vs. Lot 3**
**GMT Ratio ***	**1·02**	**0·94**	**0·92**
(95% CI)	(0·71, 1·47)	(0·67, 1·33)	(0·64, 1·31)
p-value	***0·9015***	***0·7229***	***0·6404***

* All 95% CI values for GMT ratios are within the range of [0.5 to 2.0], demonstrating that there is consistency across the three lots.

Supplementary Table 3: SARS-CoV-2 S1 protein specific IgG and IgA binding antibody responses (ELISA) and mucosal salivary (secretory) sIgA (ELISA) at Days 0 and 42, after two doses of intranasal BBV154 or intramuscular Covaxin.

		n	GMT	(95% CI)	GMT ratio (BBV154:Covaxin) Ratio	(95% CI)
**Anti-S1 IgG (serum)**						
**Day 0**	**BBV154**	481	**3675**	(3191–4232)	**1·2**	(0·9–1·6)
	**Covaxin®**	159	**3090**	(2435–3922)		
**Day 42**	**BBV154**	481	**7175**	(6490–7932)	**1·3**	(1·0–1·5)
	**Covaxin®**	159	**5689**	(4952–6537)		
**Anti-S1 IgA (serum)**						
**Day 0**	**BBV154**	481	**1978**	(1754–2230)	**1·2**	(0·9–1·5)
	**Covaxin®**	159	**1701**	(1382–2093)		
**Day 42**	**BBV154**	481	**3069**	(2794–3371)	**0.9**	(0·74–1·01)
	**Covaxin®**	159	**3537**	(3102–40356)		
**Anti-S1 secretory IgA (saliva)**						
**Day 0**	**BBV154**	58	**10·7**	(8·4–13·5)	**1·3**	(0·9–2·1)
	**Covaxin®**	22	**8·0**	(5·4–11·8)		
**Day 42**	**BBV154**	58	**12·3**	(8·7–17·4)	**1·9**	(1·1–3·0)
	**Covaxin®**	22	**6·6**	(4·6–9·5)		

Humoral IgG & IgA, and secretory IgA (sIgA) antibody titers are expressed as arbitrary ELISA units

Superiority was concluded if either the lower limit of the two-sided 95% CI for the ratio of GMTs (BBV154 GMT: Covaxin GMT) was ≥ 1.0

Supplementary Table 4: Additional Saliva IgA Data

(A) Homologous Phase II (CTRI/2021/09/036257): This study was conducted in Sept. 2021. saliva IgA titers in the BBV154 group in seronegative individuals are almost 4-fold higher on Day 42, compared to baseline titers. Participants with baseline titers of ≤ 8 of saliva IgA were considered seronegative.

	Day 0	Day 42	Fold Increase
	**Overall (GMTs)**		
BBV154	19.57 (11.5, 33.4)	30.60 (16.5, 56.6)	1.56
Placebo	29.63 (12.0, 73.0)	23.52 (6.7, 82.3)	0.79
	**Seronegative (GMTs)**		
BBV154	4.95 (4.0, 6.1)	17.80 (6.8, 46.7)	3.60
Placebo	4.0 (4.0, 4.0)	8.00 (8.0, 8.0)	1.41
	**Seropositive (GMTs)**		
BBV154	52.79 (30.8, 90.5)	45.25 (19.7, 103.8)	0.86
Placebo	47.55 (23.0, 98.4)	32.00 (6.4, 160.6)	0.67

(B) Heterologous Phase II (CTRI/2021/08/035993): This study was conducted in Aug. 2021. Saliva IgA titers of seronegative individuals in the BBV154 group are almost 5-fold higher on Day 37, compared to baseline titers. The fold increase is also 2-fold higher in BBV154 compared to COVAXIN. Participants with baseline titers of ≤ 8 of saliva IgA were considered seronegative.

	Day 0	Day 37	Fold Increase
	**Overall (GMTs)**		
BBV154	10.27 (7.5, 14.1)	33.82 (20.1, 57.0)	3.29
COVAXIN	15.50 (10.4, 23.1)	27.34 (14.8, 50.5)	1.76
	**Seronegative (GMTs)**		
BBV154	6.06 (5.0, 7.4)	27.86 (12.4, 62.7)	4.59
COVAXIN	6.35 (4.9, 8.3)	16.00 (5.0, 51.1)	2.52
	**Seropositive (GMTs)**		
BBV154	22.63 (15.9, 32.1)	45.25 (24.1, 84.9)	2.00
COVAXIN	28.76 (20.6, 40.2)	39.61 (19.0, 82.5)	1.38

Supplementary Figure 1A: Immunoglobulin Subclass Analysis.

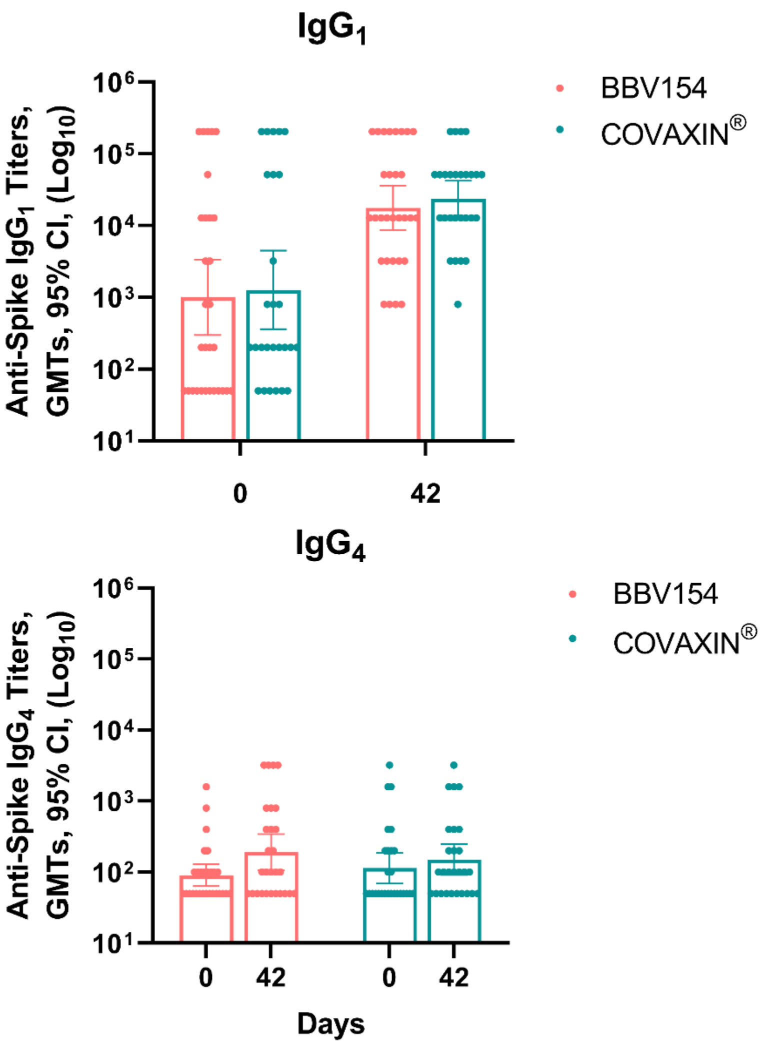

The scatter plot indicates spike-specific IgG1 and IgG4 endpoint titers, represented as Geometric mean titers at 95% Confidence intervals (GMTs, 95% CI) on Y-axis. Each dot represents an individual antibody titer. Serum samples (BBV154, n=28; Covaxin, n=27) were collected on days 0 and 42, before and after the two doses and Spike-specific IgG1 and IgG4 antibody titers were measured by ELISA. Cut-off (Mean + 3 SD) was determined by calculating the absorbance obtained at all serum dilutions (except the lower dilution, 1:50) of a known negative control (unvaccinated sera).

Supplementary *Figure 1B:* *IFN-γ Secreting T cells in Seronegative subjects*

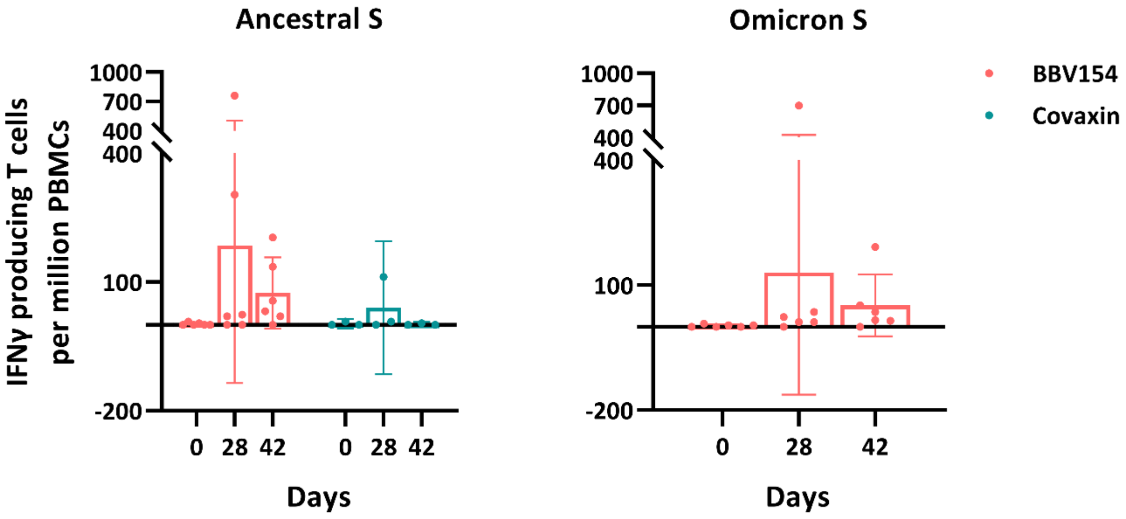

The scatter plot indicates the frequency of IFN-γ Secreting T cells in Seronegative subjects measured by ELISpot assay against ancestral and Omicron. Frequency of IFN-γ Secreting T cells per million PBMCs was shown on the Y-axis as a mean with 95% CI. Error bars indicates 95% confidence intervals. Statistical analysis was done by ANOVA (repeated measures) followed by the Tukey comparison test to compare the CMI responses observed within each group, BBV154 or Covaxin at day 28 or 42, versus Day 0. There were no significant differences between time points within each group (BBV154 or Covaxin). Further, Sidak Multiple comparison tests were used to analyze to compare BBV154 and Covaxin at different time points. There were no significant differences between BBV154 vs Covaxin at all time points. Please note that the data was not shown in the COVAXIN group against Omicron due to the non-availability of seronegative subjects. The formal size was also not shown.

Supplementary Figure 2A: Gating Strategy used to Select AIM+ SARS-CoV-2 Specific CD4⁺ T-cell Population

(A)

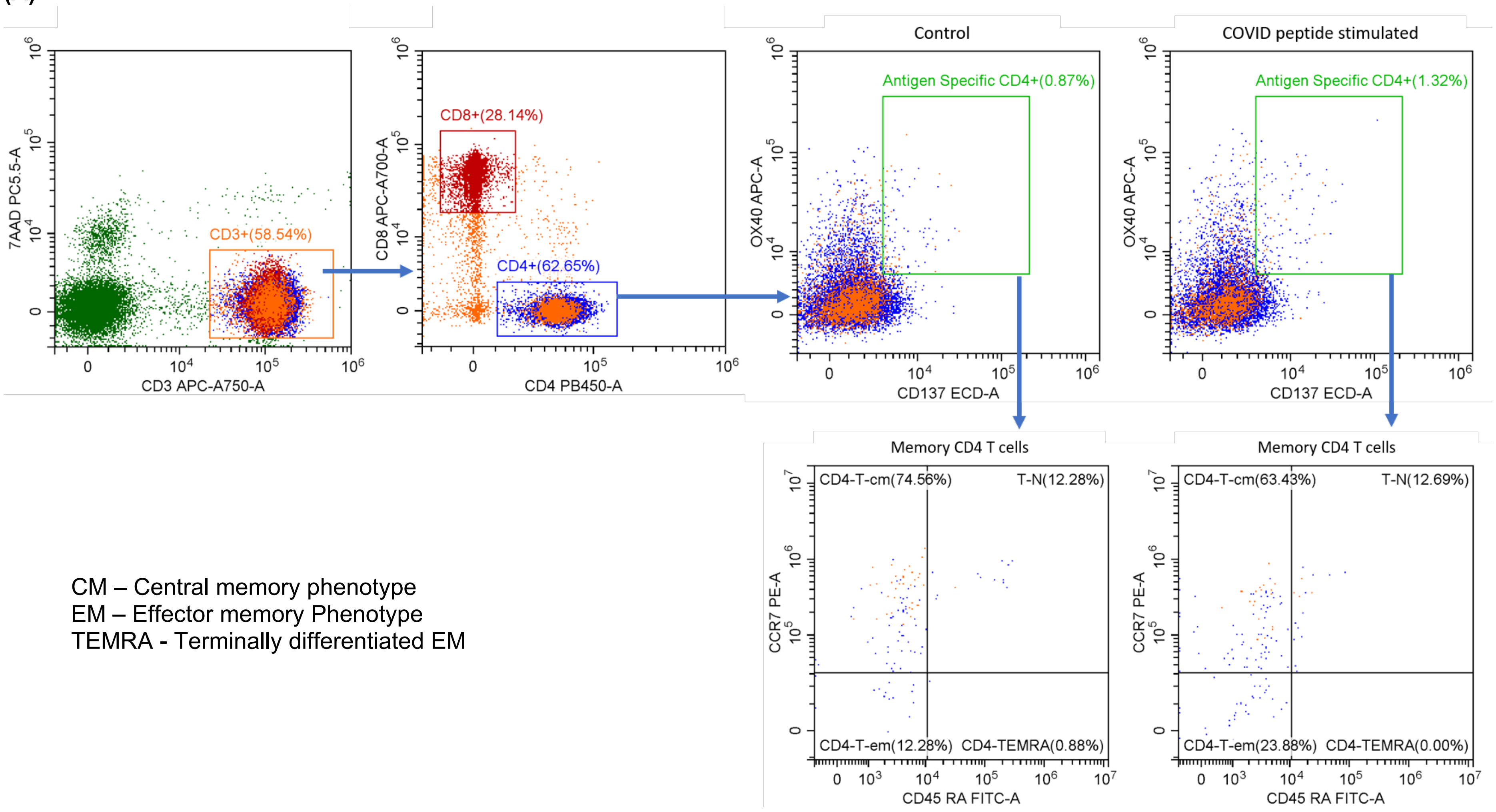

CM – Central memory phenotype
EM – Effector memory Phenotype
TEMRA - Terminally differentiated EM

Supplementary Figure 2B: Gating Strategy used to Select AIM+ SARS-CoV-2 Specific CD8⁺ T-cell Population

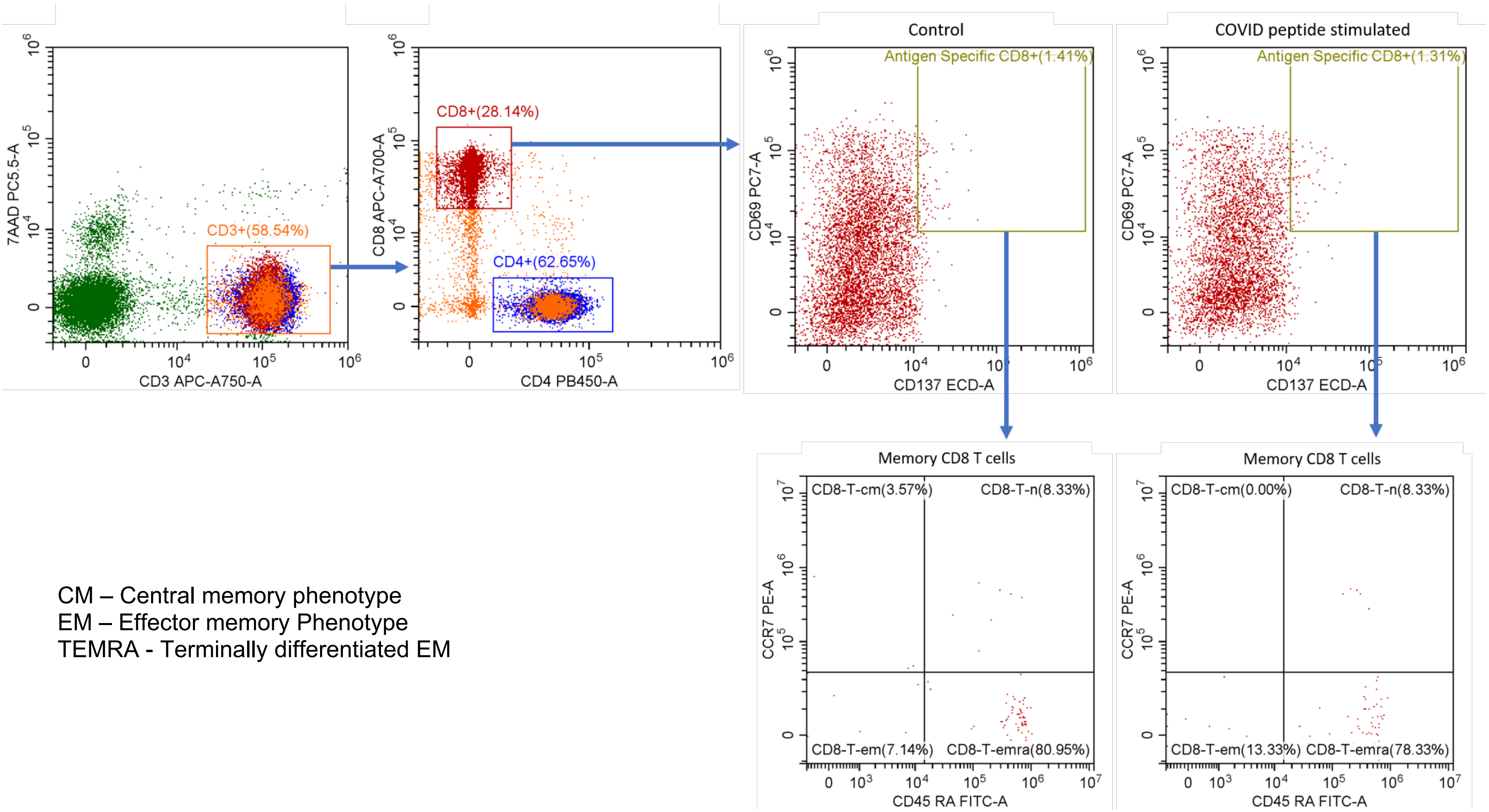

CM – Central memory phenotype
EM – Effector memory Phenotype
TEMRA - Terminally differentiated EM

Supplementary Figure 3: Memory T cell phenotype distribution

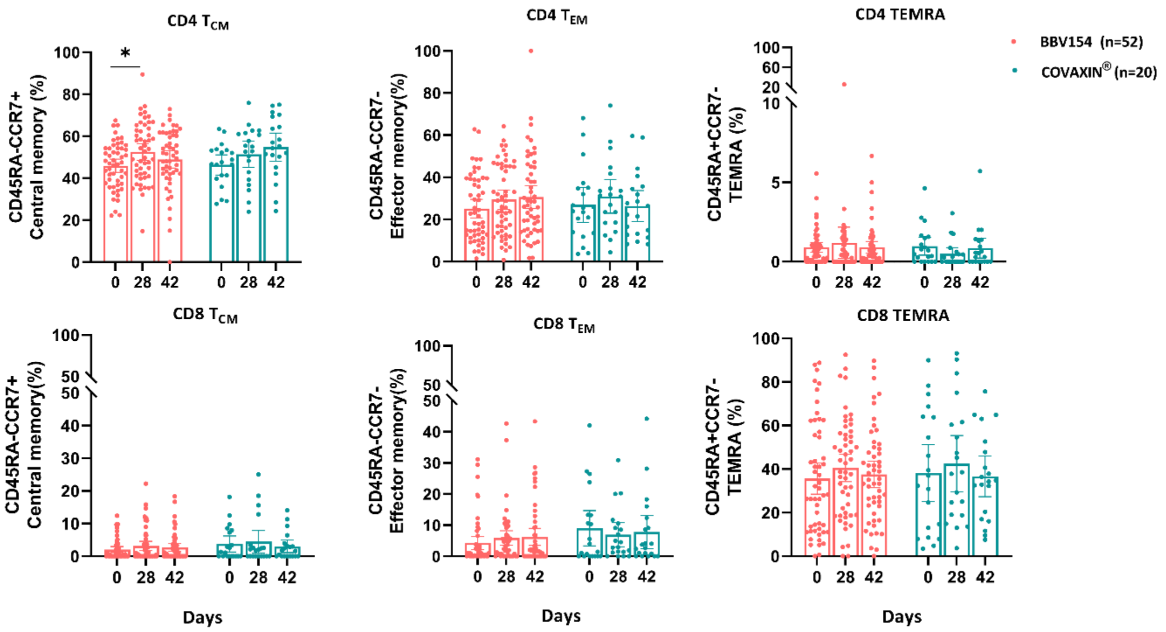

*The scatter plot represents memory T cell phenotype distribution within the AIM⁺ antigen-specific CD4⁺ and CD8⁺ T cell subsets, performed by AIM (Activation Induced Marker) assay. Percent of AIM⁺ antigen-specific CD4⁺ and CD8⁺ T cell subsets were represented as Mean with 95% CI (confidence Intervals). Error bars indicates 95% confidence intervals. Statistical analysis was done by ANOVA (repeated measures) followed by the Tukey comparison test to compare the CMI responses observed within each group, BBV154 or Covaxin at day 28 or 42, versus Day 0. There were no significant differences between time points within each group (BBV154 or Covaxin), except, where asterisk/s indicated in the graph (NS-non-significant; * p < 0·05 and ** p < 0·01). Further, Sidak Multiple comparison tests were used to analyze to compare BBV154 and Covaxin at different time points. There were no significant differences between BBV154 vs Covaxin at all time points.*

Among the AIM^+ Omicron specific $CD4^+$ T cell population, the proportions of T_{CM} ($CCR7^+CD45RA^-$) phenotype distribution is high followed by T_{EM} ($CCR7^-CD45RA^-$) compared to $CD4^+$ $CCR7^-$ $CD45RA^+$ (T_{EMRA}) phenotype. On days 0, 28 & 42, the proportion of T_{CM} was (Mean, 95% CI) 46.4% (43.2, 49.66), 53.3% (49.1-57.5), 49.4% (45.3,53.5) and 46% (41.6, 51.2), 51.4% (45.1, 57.7), 54.8% (48.0, 61.5) in BBV154 and Covaxin® groups, respectively. Whereas, the proportion of T_{EM} cells at days 0, 28 & 42 were 25.1% (20.8, 29.5), 29.4% (24.8, 34.0), 30.6% (25.1, 36.1), and 26.9% (18.6, 35.1), 31.0% (22.9, 39.0), 26.3% (19.0, 33.7), whereas proportions of T_{EMRA} cells were 0.9% (0.6, 1.2), 1.21% (0.2, 2.2), 0.9% (0.5, 1.3) and 1.0% (0.4, 1.6), 0. 5% (0.1, 0.9), 0.9% (0.2, 1.5) in both the groups.

In contrast, among the SARS-CoV-2-specific $CD8^+$ T cell population, the proportions of T_{EMRA} phenotype distribution are high compared to T_{CM} & $T_{EM.}$ On day 0, 28 & 42, the proportion of T_{CM} were 2.1% (1.2,3.0), 3.2% (1.8, 4.6), 2.7% (1.5, 3.9), and 3.8% (1.3, 6.2), 4.5% (1.0, 7.9), 3.0% (1.1, 5.0) in BBV154 and Covaxin® groups, respectively. Whereas, the proportion of T_{EM} cells, at days 0, 28 & 42 were 4.3% (2.2, 6.4), 6.0% (3.7, 8.4), 6.2% (3.5, 9.0), and 9.0% (3.3, 14.7), 7.0% (3.1, 10.9), 7.8% (2.5, 13.2), whereas proportions of T_{EMRA} cells were 35.7% (28.5, 42.8), 41.3% (35.1, 47.6), 37.6% (31.5, 43.6) and 38.2% (25.2, 51.2), 42.5% (29.6, 55.4), 36.6% (27.3, 46.0) in both the groups.

Supplementary Figure 4: IgG/IgA producing Memory B cells (MBCs)

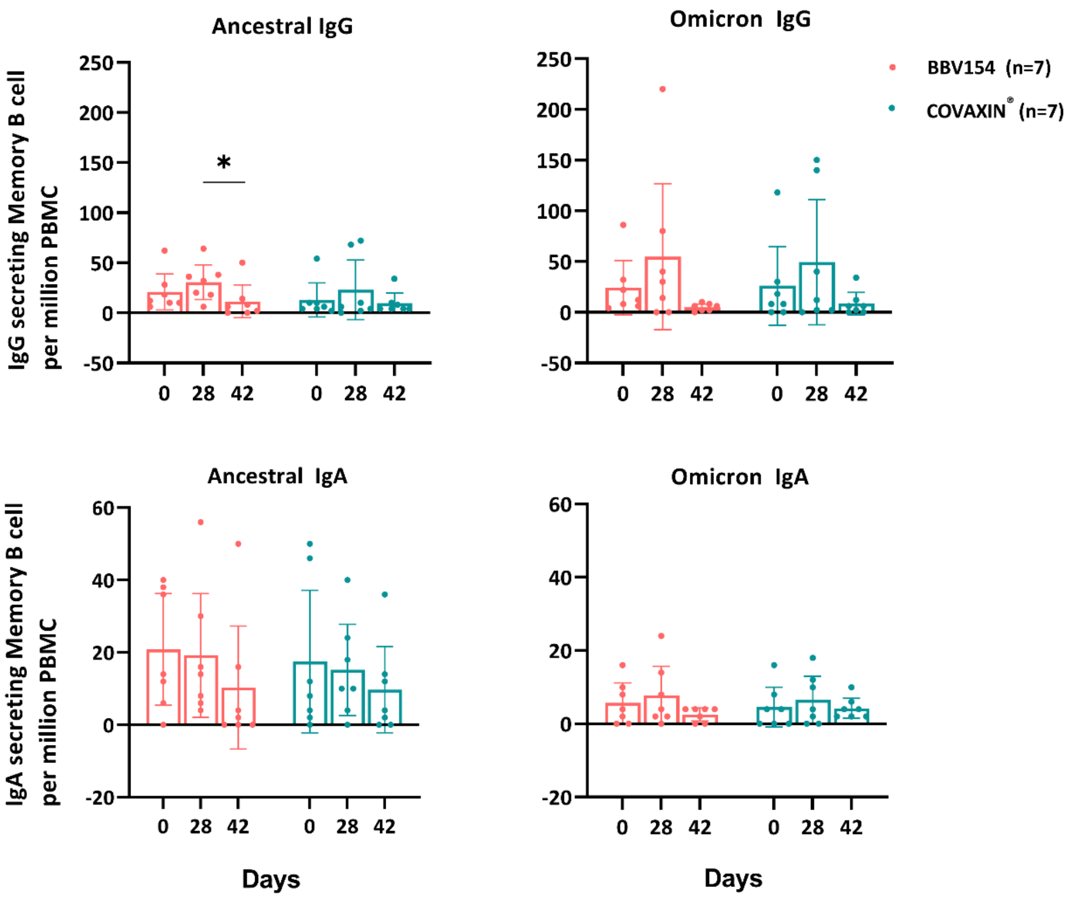

*Statistical analysis was done by ANOVA (repeated measures) followed by Tukey comparison test to compare the CMI responses observed within each group, BBV154 or Covaxin at day 28 or 42, versus Day 0. SARS-CoV-2-specific (ancestral) IgG or IgA secreting MBCs per million PBMCs were represented as Mean with 95% CI (confidence Intervals). Error bars indicates 95% confidence intervals. There were no significant differences between time points within each group (BBV154 or Covaxin), except, where the asterisk/s indicated in the graph (NS-non-significant; * p < 0·05 and ** p < 0·01). Further, Sidak Multiple comparison test was used to analyze to compare BBV154 and Covaxin at different time points. There were no significant differences between BBV154 vs Covaxin at all time points.*

SARS-CoV-2-specific (ancestral) IgG secreting MBCs per million PBMCs, (Mean, 95% CI) at day 0, 28 & 42 were 20·9 (2.8, 38.9), 30·6 (13.4, 47.8), 11·4 (-5.0, 27.8) and 12·9 (-4.0, 29.9), 23·1 (-6.6, 52.9), 9·7 (-0.4, 19.9) in BBV154 and Covaxin® groups, respectively. Similarly, omicron specific IgG secreting MBCs per million PBMCs, (Mean, 95% CI) at day 0, 28 & 42 were 24·3 (-2.5, 51.1), 54·9 (17.2, 126.9), 4·9 (1.5, 8.2) and 26·0 (-12.8, 64.8), 49·4 (-12.3, 111.2), 8·6 (-2.5, 19.7) in BBV154 and Covaxin® groups, respectively.

Similarly, SARS-CoV-2-specific (ancestral) IgA secreting MBCs per million PBMCs, (Mean, 95% CI) at day 0, 28 & 42 were 20·9 (5.4, 36.3), 19·1 (2.1, 36.2), 10·3 (-6.7, 27.3) and 17·4 (-2.3, 37.1), 15·1 (2.6, 27.7), 9·7 (-2.2, 21.6) in BBV154 and Covaxin® groups, respectively. Similarly, omicron specific IgA secreting MBCs per million PBMCs, (Mean, 95% CI) at day 0, 28 & 42 were 5·7 (0.2, 11.2), 7·7 (-0.2, 15.7), 2·6 (0.8, 4.3) and 4·6 (-0.8, 10.0), 6·6 (0.2, 13.0), 4·3 (1.6, 7.0) in BBV154 and Covaxin® groups, respectively.

Supplementary Figure 5: Nasal delivery of BBV154 is less likely to elicit vector specific antibodies

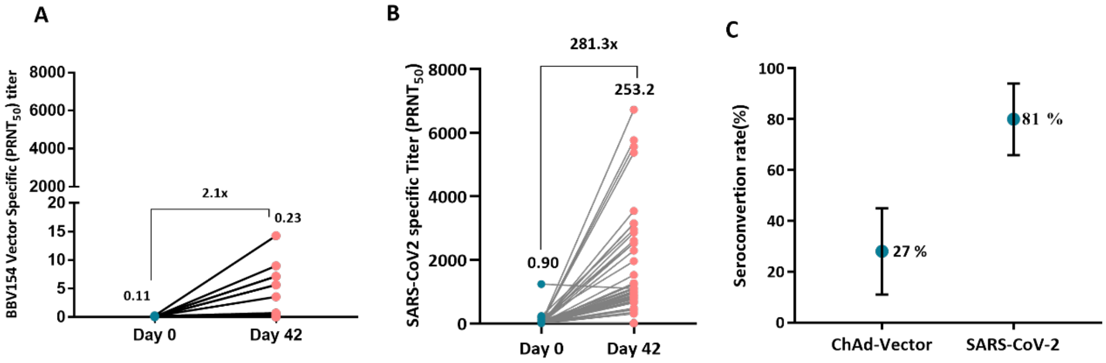

---

*BBV154 vector-specific antibody titer $PRNT_{50}$ (A) and SARS-CoV-2 neutralizing ($PRNT_{50}$) (B) were analyzed (*Geometric mean titers (95% CI) *at Days 0 (baseline), Day 42, four weeks after the booster dose of BBV154 (n=64).* Error bars represent 95% confidence intervals. Vector specific neutralization antibody titers are very much less at Day 42 with a GMT 0.2 (0.16, 0.32), [2.1-fold higher than day 0, with a GMT, 0.11 (0.099, 0.12)], Whereas, SARS-CoV-2 specific neutralization titers at day 42 were 281 fold higher than day 0 [Day 42 GMTs 253.2 (103, 639 *vs* Day 0 GMTs 0.9 (0.41, 1.98)]. It was also noticed that 27% of the participants showed 4-fold seroconversion against the ChAd vector, whereas 81% of the participants seroconverted against SARS-CoV-2 virus.
